# Corrigendum to “The Analysis of Biomechanical Properties of Proximal Femur after Implant Removal”

**DOI:** 10.1155/2020/3629465

**Published:** 2020-07-16

**Authors:** Jae Hyuk Yang, Tae Gon Jung, Arjun Rupanagudi Honnurappa, Jae Min Cha, Chang Hwa Ham, Tae Yoon Kim, Seung Woo Suh

**Affiliations:** ^1^Scoliosis Research Institute, Department of Orthopedics, Korea University Medical College, Guro Hospital, Guro 2-dong, Guro-gu, Seoul 152-703, Republic of Korea; ^2^Osong Medical Innovation Foundation, Medical Device Development Center, Cheongju 363-951, Republic of Korea; ^3^The Division of Biological Sciences at The University of Chicago, 5801 South Ellis Avenue Chicago, IL 60637, USA; ^4^Jung-Hwa Girls High School, San 105 Beomeo-4-dong Sunsung-gu, Daegu 706-819, Republic of Korea

In the article titled “The Analysis of Biomechanical Properties of Proximal Femur after Implant Removal,” [1] it is incorrectly stated that the study received funding from the Korea Healthcare Technology R&D project. The authors apologise for this error and have clarified that no specific funding was obtained for this research.
